# Colistin Resistance Onset Strategies and Genomic Mosaicism in Clinical *Acinetobacter baumannii* Lineages

**DOI:** 10.3390/pathogens10111516

**Published:** 2021-11-20

**Authors:** Viviana Cafiso, Stefano Stracquadanio, Veronica Dovere, Flavia Lo Verde, Alessandra Zega, Giuseppe Pigola, Simona Barnini, Emilia Ghelardi, Stefania Stefani

**Affiliations:** 1 Department of Biomedical and Biotechnological Sciences, University of Catania, 95123 Catania, Italy; s.stracquadanio@unict.it (S.S.); flavia.loverde@hotmail.it (F.L.V.); alessandra.zega@libero.it (A.Z.); gpigola@gmail.com (G.P.); stefania.stefani@unict.it (S.S.); 2 Department of Translational Research and New Technology in Medicine and Surgery, Azienda Ospedaliero-Universitaria Pisana, University of Pisa, 56126 Pisa, Italy; veronicadovere@gmail.com (V.D.); emilia.ghelardi@med.unipi.it (E.G.); 3 Bacteriology Unit of Azienda Ospedaliero-Universitaria Pisana, 56126 Pisa, Italy; barninis@gmail.com

**Keywords:** *A. baumannii*, colistin resistance stability, genomic mosaicism, phylogenetic lineages

## Abstract

The treatment of multidrug-resistant Gram-negative infections is based on colistin. As result, COL-resistance (COL-R) can develop and spread. In *Acinetobacter baumannii*, a crucial step is to understand COL-R onset and stability, still far to be elucidated. COL-R phenotypic stability, onset modalities, and phylogenomics were investigated in a clinical *A. baumannii* sample showing a COL resistant (COL^R^) phenotype at first isolation. COL-R was confirmed by Minimum-Inhibitory-Concentrations as well as investigated by Resistance-Induction assays and Population-Analysis-Profiles (PAPs) to determine: (i) stability; (ii) inducibility; (iii) heteroresistance. Genomics was performed by Mi-Seq Whole-Genome-Sequencing, Phylogenesis, and Genomic Epidemiology by bioinformatics. COL^R^ *A. baumannii* were subdivided as follows: (i) 3 *A. baumannii* with stable and high COL MICs defining the “homogeneous-resistant” onset phenotype; (ii) 6 *A. baumannii* with variable and lower COL MICs displaying a “COL-inducible” onset phenotype responsible for adaptive-resistance or a “subpopulation” onset phenotype responsible for COL-heteroresistance. COL-R stability and onset strategies were not uniquely linked to the amount of LPS and cell envelope charge. Phylogenomics categorized 3 lineages clustering stable and/or unstable COL-R phenotypes with increasing genomic complexity. Likewise, different nsSNP profiling in genes already associated with COL-R marked the stable and/or unstable COL-R phenotypes. Our investigation finds out that *A. baumannii* can range through unstable or stable COL^R^ phenotypes emerging via different “onset strategies” within phylogenetic lineages displaying increasing genomic mosaicism.

## 1. Introduction

In the last twenty years, *Acinetobacter baumannii* has become a major threat not only as an important pathogen associated with nosocomial and community infections at various body sites, including the bloodstream, respiratory tract, urinary tract, surgical sites, and wounds [1], but also because it poses a huge challenge in clinical settings due to its intrinsic and acquired antimicrobial resistance. The development of multidrug resistance (MDR) in *A. baumannii* is due to genomic mutations, acquired antimicrobial resistance genes, or modifications in the expression of some genes [2]. Because of the significant increase in the isolation of carbapenem-resistant *A. baumannii* (CR-*A. baumannii*) and, until recently, the lack of active drugs against this microorganism, colistin (COL) has been used to treat CR-*A. baumannii* infections despite its nephrotoxicity, representing, in combination, a last-line therapy. Eventually and not unexpectedly, the emergence of colistin-resistant (COL^R^) *A. baumannii* was reported worldwide [3] and it is caused by mutations or altered expression of diverse genes—mainly *pmr* and *lpx* genes [4] as well as *gal*U [5]—or the acquisition of the plasmidic gene *mcr* [6]. Nonetheless, colistin resistance (COL-R) is still low or variable across countries, while, as reported by Karokostantis (2020), the prevalence of COL heteroresistance seems to be more common [3,7,8]. Heteroresistance is usually defined as the presence of subpopulations with MIC values higher (variably defined as equal to or more than two- to eight-fold) than the MIC in the main population, is often associated with the previous use of the drug, and represents major trouble in clinical settings as resistant subpopulations can emerge during treatment, resulting in treatment failure. Colistin heteroresistance in *A. baumannii* is even more alarming as it was detected even in isolates without prior exposure to colistin, suggesting that it could be an inherent characteristic of this species [8,9]. Moreover, a concentration of antibiotic >128 mg/L was shown to be necessary to prevent the onset of colistin heteroresistant *A. baumannii* mutants, as previously reported [10]; this is much higher than colistin serum concentrations (median 2.36 mg/L) associated with approximately 50% toxicity rates [10]. Adding to the complexity in fully understanding the COL^R^ *A. baumannii* threat, there are cases where COL minimum inhibitory concentration (MIC) values vary over time for the same *A. baumannii* strain, ranging from values lower than the susceptibility cut-off to higher values [11] even in the absence of heteroresistant subpopulations.

As already stated, different studies showed that COL heteroresistance in *A. baumannii* is common, but the real frequency rate is not determined [12], probably due to the absence of easy-to-perform tests, ranging from 18% to 100% of COL heteroresistant clinical strains. Furthermore, studies do not usually perform either resistance induction assays or MIC tests on strains cultured for different days [13,14]. These lack of analyses could lead to a misunderstanding in the detection of the COL heteroresistance, as it may result in underestimated or overestimated since the induction assays could promote the raise of COL^R^ subpopulation, whereas culturing resistant strains for a long time could result in the loss of COL-R. 

Although some studies have already investigated the presence of well-known mutations in the genes responsible for COL-R in resistant and heteroresistant *A. baumannii* strains [3,15,16,17], to the best of our knowledge no one has ever analyzed their whole genomes to group them based on their similarities and differences. Herein, we conducted a phenotypic and genomic characterization as well as investigated the phylogenetic relationship in *A. baumannii* samples displaying different levels of colistin resistance and heteroresistance, suggesting three different strategies that *A. baumannii* can use to become resistant.

## 2. Results

### 2.1. COL MIC 

In 10 repeated MIC assays, the 1R, 2R, and 3R strains showed a stable and high COL MIC of 128 mg/L defining the "homogeneous-resistant" onset phenotype, and hence Full-Resistance. Contrarily, in the same 10 times repeated MIC tests, the second group of *A. baumannii* strains showed unstable COL MICs, as follows: COL MIC values ranging from 1 to 64 mg/L, with the most frequent value being 8 mg/L in 4R; from 2 to 16 mg/L, with the most frequent value being 16 mg/L in 5R; from 2 to 128 mg/L, with most frequent values being 64 and 2 mg/L in 6R and 7R, respectively; from 2 to 32 mg/L, with the most frequent value being 32 mg/L in 8R; and from 1 to 128 mg/L with 4 mg/L as the most frequent value in 9R (Table 1). 

### 2.2. COL Resistance Induction

Colistin resistance induction assays showed a drug-inducible COL-R phenotype reported as “COL-inducible” onset phenotype in 6R and 8R. The induced 6R and 8R variants, having a stable COL MIC > 256 mg/L, were considered as having Adaptive-Resistance. Strains 4R, 5R, 7R, and 9R showed no growth at colistin concentrations ≥ 2 mg/L (Table 1). 

### 2.3. Hetero-Resistance

**Figure 1:**
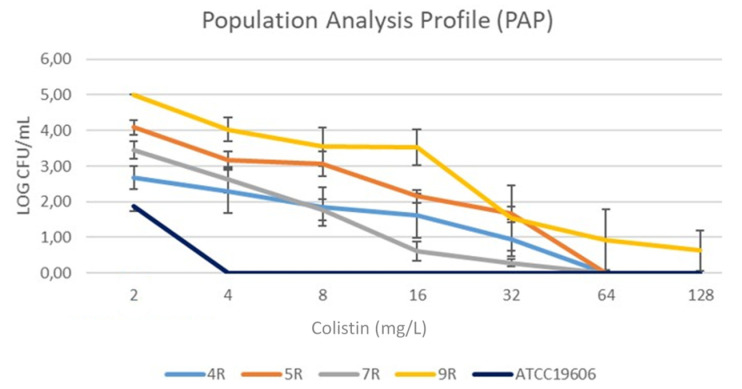
PAPs for the 4R, 5R, 7R, and 9R *A. baumannii* strains.

**Figure 2:**
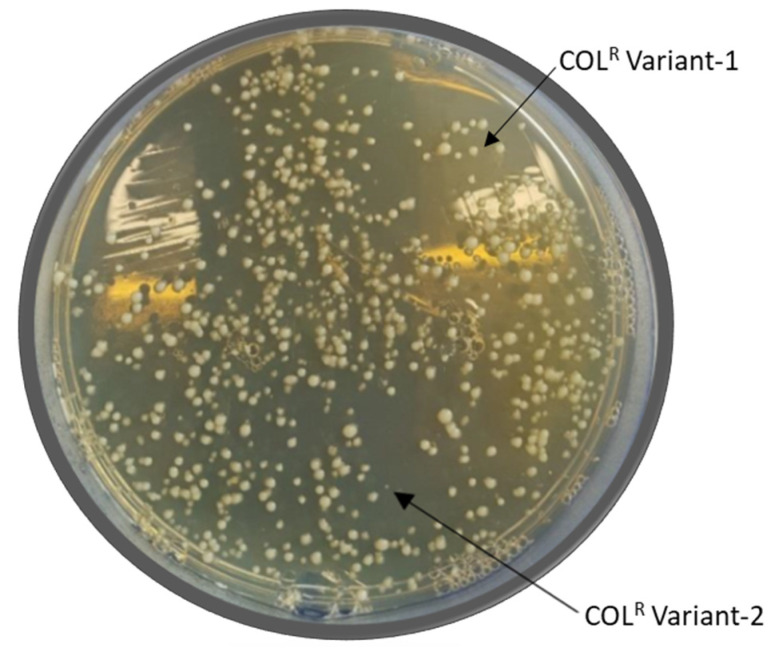
Representative Morphologies of the COL^R^ *A. baumannii* variants detected in 4R, 5R, and 7R detected on 32 mg/L COL-agar plates and 128 mg/L COL-agar plates in 9R strains.

COL Population Analysis Profiles (PAPs) revealed the presence of subpopulations able to grow up to COL 64 mg/L in 4R, 5R, and 7R, and up to 128 mg/L in 9R *A. baumannii*, as shown in Figure 1. Furthermore, the presence of two morphologically different subpopulations, named COL^R^ variant-1 and COL^R^ variant-2, in COL 32 mg/L agar plates in 4R, 5R, and 7R as well as in COL 128 mg/L agar plates in 9R was also detected (Table 1), (Figure 2). This phenomenon was referred to as “heteroresistance” and identified the “subpopulation” onset phenotype. The COL^R^ variant-1 and COL^R^ variant-2 showed a 2 to 6-fold increase in COL MIC in 4R, 5R, 7R, and a 1- fold COL MIC increase in 9R (Table 1). 

### 2.4. LPS Quantification and Cell-Envelope Charge

Lipopolysaccharide (LPS) and cell envelope net positive charge quantification tested in all strains on a normalized total number of bacterial cells are shown in Table 1. 

### 2.5. Phylogenetic Tree and Genomic Typing 

**Figure 3:**
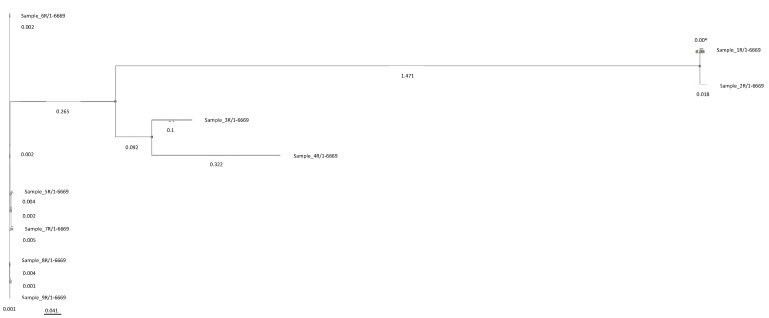
Phylogenetic tree of *A. baumannii* strains referred to RefGen *A. baumanni* ACICU, realized with the CSI Phylogeny tool.

Genomic Phylogeny (gPhyl) recognized three different lineages, as shown by the Whole Genome SNP-based phylogenetic tree (Figure 2). 5R, 6R, 7R, 8R, and 9R *A. baumannii* were grouped in gPhyl lineage-I, 3R, and 4R in gPhyl lineage-II and 1R and 2R in gPhyl lineage-III (Figure 3). gPhyl lineage-I only showed unstable COL^R^ *A. baumannii*, gPhyl lineage-II had one stable and one unstable COL^R^ *A. baumannii*, whereas gPhyl lineage-III only included stable COL^R^ *A. baumannii*. 

According to the Oxford University database, the gPhyl lineage-I *A. baumannii* strains belonged to the ST-1808 and ST-348, KL-9, and OCL-1 types; the gPhyl lineage-II *A. baumannii* strains belonged to diverse MLSTs (ST-1816 and ST-195 for 3R and ST-218 for 4R) and OCL-1 type, and different KL-3 e and KL-28, respectively; the gPhyl lineage-III *A. baumannii* belonged to ST-1839 and ST-218 for 1 R, having two gdhB alleles with 100% identity and coverage, along with ST-1839 for 2R, K-22, and OCL-3 types. According to the Pasteur Institute database, all *A. baumannii* strains were assigned to ST-2, except for 1R, belonging to ST-187, a ST-2 Single Locus Variant (SLV) (Table 2).

All *A. baumannii* lacked the CRISPR/Cas systems, with the exception of 3R carrying 3 questionable (not confirmed) CRISPR regions with 1 spacer, and 6R having 2 questionable (not confirmed) CRISPR regions with 1 spacer, with both strains lacking Cas genes. Abundant mobile genetic elements including Insertion Sequences and Transposons were found in all *A. baumannii*, whereas the vB_AbaS_TRS1 and the Bφ_B1251 prophages were only found in 1R, 2R, 3R, and 4R. All strains were assigned to the genomic macrorestriction profile “A”, being indistinguishable by PFGE (Table 2).

### 2.6. Resistomes and nsSNPs in Genes Previously Associated to COL-R Mechanisms

Resistomics showed the acquisition or loss of genes responsible for antimicrobial resistance as reported in Table 2. Based on these data, 4 resistome-types were identified: resistome-type I, characterized by blaADC-25, blaOXA-23, blaOXA-82, aadA2 ant(2″)-Ia; aph(3′)-Via, sul1, detected in 1R and 2R (gPhyl lineage-III); resistome type-II -blaADC-25; blaOXA-23; blaOXA-66; blaTEM-1D, aph(3″)-Ib, aph(3′)-Ia, aph(6)-Id; armA, sul2, mphE, msrE, tetB-in 3R (gPhyl lineage-II); resistome type-III-blaADC-25; blaOXA-23, blaOXA-66, aph(3″)-Ib, aph(6)-Id, tetB-in 4R (gPhyl lineage-II); resistome-type IV—blaADC-25, blaOXA-66, blaOXA-72, aac(6′)-Ip, aph(3″)-Ib, aph(6)-Id, armA, sul1, sul2 (not always present), mphE, msrE, tetB—in 5R, 6R, 7R, 8R, and 9R (gPhyl lineage-I). No mcr-genes were detected in the unstable and stable COL^R^ *A. baumannii*.

Genomic Single Nucleotide Polymorphisms (SNPs) mapping on *A. baumannii* ACICU RefGen returned different nsSNPs in genes previously associated with COL-R mechanisms in *A. baumannii* and other Gram-negative species. 

In detail, all unstable members had a characterizing moderate impact effect (MI) *gal*U nsSNP determining the I245T AminoAcid (AA) change, whilst only 3 out of 6 showed *pmrB* nsSNPs. All stable COL full resistant members had diverse MI nsSNPs in both *gal*U and *pmr*B. Furthermore, all the stable COL full resistant members had the *gal*U nsSNP determining the I273V associated, in two strains, to the Q140L, along with two AA changes in PmrB (P170L and A138T) and one in LpxA (N5V) (Table 1).

No nsSNPs were detected in *pmr*AC, *lpx*DCB, and *pro*B in all *A. baumannii* on *A. baumannnii* ACICU RefGen mapping.

## 3. Discussion

The outbreak of antibiotic resistance and the emergence of MDR *A. baumannii* highlight the need to understand how resistant strains emerge and spread. The overuse and inappropriate consumption of colistin strongly correlate with the emergence of COL^R^ *A. baumannii* [18]. 

Colistin resistance in *A. baumannii* has been reported worldwide, with the highest resistance rates in Asia and then in Europe [19]; however, the rate of colistin heteroresistance in *A. baumannii* turned out to be higher than that of resistance [9,20,21,22,23].

In this context, we investigated the stability of COL-R phenotypes in *A. baumannii* phenotypically outlining the different onset strategies and genomically characterizing the phylogenetic lineages having different “stability and onset phenotypes”.

Our data, for the first time, described the phenomenon of the variable behavior of the COL-R phenotype in a group of clinical *A. baumannii*. Our observations of the MIC values, repeated several times, lead us to conclude that a stable COL-R phenotype was exhibited only in 3 out of 9 *A. baumannii*. In *A. baumannii,* high levels of COL-R (MIC 128 mg/L) were detected within the entire bacterial population emerging, thus, with a “homogeneous-resistant” onset phenotype, and hence considered as “Full-Resistance”. The Full-Resistant *A. baumannii* were already described and our data was in agreement with previously published data and well-characterized strains [2,24]. Contrarily, the COL-R phenotype instability was described by our group, for the first time, in 6 *A. baumannii* showing mainly variable and lower COL MICs ranging from 1 to 128 mg/L. This original observation opens a new perspective in the understanding of the COL-R emergence strategies under colistin pressure and could be crucial for the clinical implication in the treatment of severe MDR Gram-negative infections. 

The unstable COL-R phenotype was related by our group to two different onset strategies, i.e., a “COL-inducible” onset phenotype, considered as an “Adaptive Resistance” -in agreement to prior observations [25,26]— and a “Subpopulation” onset phenotype considered as “Heteroresistance” previously described by another group [27]. In our *A. baumannii*, the “COL-inducible” onset-phenotype emerged via an induction mechanism related to a constant long term colistin exposure, whilst the “Subpopulation” onset-phenotype emerged spontaneously in a subset of populations with higher COL-R levels, detectable as morphological variants with different COL MICs, exclusively in the presence of high COL concentrations (i.e., ≥COL 32/128 mg/L agar plates). 

No correlation was found between the COL-R stability and the different onset strategies with LPS amount and cell-envelope charge. Therefore, we hypothesized that LPS alterations and perturbations in the negative envelope charge can exist as single or coexisting features, although in a strain-dependent manner and independently to the stability of the COL-R phenotype. In our opinion, the co-occurrence of a pool of the two factors might affect the envelope structure and charge both directly and/or indirectly.

Genomic Phylogeny arises firstly new consideration about the dynamics of COL^R^ stable phenotype emergence, leading us to evidence that the progression from an unstable and stable phenotype appeared related to specific phylogenetic lineages characterized by high genomic mosaicism due to acquisition or loss of MGEs or Phages. The Genomic Phylogeny clustered our clinical *A. baumannii* sample in 3 phylogenetic lineages and the phylogram indicated gPhyl lineage-I as the common ancestor of gPhyl lineage-II and gPhyl lineage-III. Interestingly, gPhyl lineage-I only included unstable COL^R^ *A. baumannii* with hetero- or adaptive COL-R, gPhyl lineage-II clustered one stable, homogeneous, COL Full-Resistant *A. baumannii* together with one unstable, heteroresistant COL^R^ *A. baumannii*, whereas gPhyl lineage-III only included stable, homogeneous Full-Resistant COL^R^ *A. baumannii*. Notably, the analysis of the evolutionary relationship of the gPhyl lineages clearly showed that gPhyl lineage-II, including unstable and stable COL-R phenotypes, may be considered as a “hybrid lineage” branching out from gPhyl lineage-I as a midpoint in the evolution of these strains, whereas gPhyl lineage-III, last differentiated, with only stable Full-Resistant COL^R^ *A. baumannii*, may be considered as an endpoint in the COL^R^ *A. baumannii* strain evolution, in agreement with the progression of COL-R stability acquisition. 

This described phylogenetic evolution for the first time supports the hypothesis that the acquisition of a stable, homogeneous, full COL-R moves its first steps in the subpopulation or the adaptive onset strategy that progresses until reaching stability and homogeneity. 

Furthermore, the gPhyl lineage-I *A. baumannii* strains belonged to the ST-1808 and ST-348, KL-9, OCL-1, and resistome-IV type; the gPhyl lineage-II *A. baumannii* strains belonged to diverse MLSTs (ST-1816 and ST-195 for 3R and ST-218 for 4R), KL-3 or KL-28, OCL-1, and resistome-II or III type; the gPhyl lineage-III *A. baumannii* strains belonged both to the ST-1839, KL-22, OCL-3, and resistome-I type, showing that belonging to a specific gPhyl lineage is related to intrinsic clonality of its members. By contrast, the mobilome and prophage profile showed a mosaic of mobile genetic elements (MGEs) and phages ranging from a higher number of transposable elements, such as 6-8 different insertion sequence elements (ISs) and one transposon (Tn6207) in gPhyl lineage-I unstable COLR A. baumannii, to a lower IS set (3–6) and one transposon (*Tn*6080 or *Tn*2006) associated to one and/or two different phages (vB_AbaS_TRS1 and the Bφ_B1251) as in gPhyl lineage-II and III. The MGE and phage toolbox showed dynamism inside the highly clonal conserved *A. baumannii* genomes, resulting in increased variability and complexity related to the transition from an unstable to a stable COL-R phenotype, besides being responsible for genome evolution and high antimicrobial adaptation ability. Furthermore, resistomics highlighted that the gPhyl lineage-III—with its stable, homogeneous and full resistance—surprisingly appeared short of acquired resistance determinants, such as the genes involved in tetracycline and macrolide resistance, with respect to the other two mainly unstable phylogenomic lineages. 

Furthermore, we can speculate that genetic variations in genes already related to polymyxin B (PB) and COL resistance seem, for the first time, related to the progression towards a stable COL^R^ phenotype. New mutations in *gal*U— encoding an enzyme related to polymyxin B (PB) resistance in *Proteus mirabilis* [28]— and in *pmr*B, involved in the COL-R in *A. baumannii* [4] and Enterobacterales [29], were found out in the sample. New nsSNPs, computationally predicted affecting protein functionality, were mapped in GalU ligand binding site I245T in the unstable COL^R^ and I273V in the stable one. GalU-UTP-glucose-1-phosphate uridylyltransferase is a key enzyme involved in the pathway of the lipopolysaccharide biosynthesis, which is part of outer membrane biogenesis [30]. *P. mirabilis* knockout mutants in GalU were found to be extremely sensitive to PB, presumably because of changes in LPS [29]. Likewise, no mcr-mediated resistance mechanisms are implicated in the stability of COL-R phenotypes as mcr-genes were not found [6].

Of note, the genomic snapshot of the three different “COL-R onset phenotypes showed that the progression from COL-R instability to stability seems to be associated with a gradual genome evolution from “low-complexity” to “high-complexity” lineages through a pool of complex rearranged genomes.

In conclusion, our investigation finds out a new correlation between the in vitro COL-R stability and onset strategies as well as the membership to specific phylogenetic lineages evolving through complex genomic rearrangements involving whole-genome SNPomes, MGEs, Resistomes, Phage Profiling.

Our data demonstrated, for the first time, the association between the shift to unstable to stable COL-R phenotypes and increasing genomic mosaicism within the intrinsic high clonality of COL^R^ *A. baumannii.* Likewise, new mutations putatively affecting the stability and functionality of GalU and PmrB can speculatively affect the dynamics of the COL-R stability acquisition.

## 4. Materials and Methods

### 4.1. Bacterial Strains

Nine clinical Extensively Drug-Resistant (XDR) *A. baumannii*, named 1-9R, were isolated from patients treated with sodium colistithemate for a long time, 7–14 days, at the Catania and Pisa University Hospitals (Table 3) 

The strains were grown in McConkey agar plates (Oxoid, UK) at 37 °C for 18 h and identified by Matrix-Assisted Laser Desorption Ionization–Time of Flight (MALDI-TOF) [31] mass spectrometry (Bruker Daltonics, Billerica, MA, USA). Antibiotypes were determined by the Phoenix system (Becton, Dickinson and Company, MD, USA) [32] and the results were confirmed by the Sensititre system (Thermo Fisher Scientific, Waltham, MA, USA) [33]. The strains were stored in glycerol at −80 °C until further analysis.

### 4.2. COL Minimum Inhibitory Concentrations (MICs)

Colistin MICs were determined by microdilution assay using sulfate colistin ≥ 15,000 U/mg (Sigma-Aldrich, Castle Hill, New Galles, Australia) in cation-adjusted Muller-Hinton broth (BBLTM MHBCA II; Becton, Dickinson and Company, MD, USA) as recommended by the Clinical and Laboratory Standards Institute (CLSI) [34] and the EUCAST guidelines. The results were interpreted according to the current colistin breakpoints for *A. baumannii* reported in EUCAST v9.0 [35] (≤2 mg/L for COL-susceptibility (COL-S) and >2 mg/L for COL-resistance (COL-R)). *Pseudomonas aeruginosa* ATCC27853 and *Escherichia coli* ATCC25922 were used as control strains for these assays. MIC assays were repeated 10 times almost every week for three months.

### 4.3. COL Resistance Induction Assay

COL-R induction assays were performed by growing daily *A. baumannii* strains at COL concentrations gradually doubled from COL 0.5 mg/L to COL 32 mg/L in Brain-Heart Infusion Broth (BHIB-Oxoid, Basingstoke, UK). Then, colistin resistance was re-evaluated by plating cultures in COL 16 mg/L agar-plates and the COL MICs were re-evaluated as described above. Each COL-R induction assay was performed in three biological replicates.

### 4.4. COL Hetero-Resistance Detection

Population Analysis Profiles (PAPs) were defined to evaluate the presence of *A. baumannii* subpopulations with a diverse rate of COL-R [9,36]. PAPs were performed by plating 50 μL of a 10^8^ CFU/mL bacterial suspension in Mueller–Hinton (MH) agar containing serial dilutions (2–128 mg/L) of sulfate colistin. After 48 h of incubation at 37 °C, the presence of “differential rate” COL-R subpopulations were evaluated. *A. baumannii* ATCC19606 was used as the control strain. For each subpopulation, “differential rate” COL-R detection was performed in triplicate.

### 4.5. Surface Charge Determination 

The cytochrome *c* binding assay was performed as a surrogate measure of the relative net positive surface charge of the strain as previously described [37,38]. Briefly, cells were grown overnight in TSB media to an OD_575_ = 10 (2 × 10^10^ CFU/mL), washed with 20 mM MOPS buffer (pH 7.0) three times, and re-suspended in the same buffer at OD_575_ = 10 (2 × 10^10^ CFU/mL). Cells were incubated with 0.5 mg/mL cytochrome *c* for 10 min and the amount of cytochrome *c* remaining in the supernatant was determined spectrophotometrically at OD_530_ nm. The greater the amount of unbound cytochrome *c* detected in the supernatant, the higher the net positive charge of the bacterial surface. Data were converted and expressed as the mean (±SD) amount of bound cytochrome *c*. At least three independent runs were performed on separate days.

### 4.6. Direct LPS Quantification

LPS was extracted by the hot phenol-water method as previously described [39,40]. Briefly, each *A. baumannii* culture was grown up to OD_650_ = 10 (2 × 10^10^ CFU/mL) in Cation adjusted Mueller Hinton broth, and cells were harvested by centrifuging the tubes at 10,000× *g* for 5 min. The pellets were washed twice in PBS (pH = 7.2, 0.15 M) containing 0.15 mM CaCl_2_ and 0.5 mM MgCl_2_, then they were resuspended in 10 mL PBS and sonicated for 10 min on ice. Treatments with proteinase K (100 µg/mL) at 65 °C for 1 h, and with DNase (20 µg/mL) and RNase (40 µg/mL) incubating the pellet at 37 °C overnight in the presence of 1 µL/mL 20% MgSO_4_ and 4 µL/mL chloroform, were performed prior to extraction step to eliminate contaminating protein and nucleic acids. Subsequently, an equal volume of hot (65–70 °C) 90% phenol was added to the mixtures followed by vigorous shaking at 65–70 °C for 15 min. Suspensions were then cooled on ice, transferred to 1.5 mL polypropylene tubes, and centrifuged at 8500× *g* for an additional 15 min. Supernatants were transferred to 15 mL conical centrifuge tubes and phenol phases were re-extracted using 300 µL dH_2_O. Sodium acetate at 0.5 M final concentration and 10 volumes of 95% ethanol were added to the extracts and samples were stored at −20 °C overnight to allow LPS precipitation. On the last day, tubes were centrifuged at 2000× *g* 4 °C for 10 min and the pellets were resuspended in 1 mL dH_2_O. LPS absorbance at OD_259_ was measured on the same day in a spectrophotometer (Thermo Scientific™ GENESYS 10S UV-Vis, Waltham, MA, USA), and the quantification was obtained by comparison with a standard curve made with scalar dilutions of extra pure *E. coli* LPS (Sigma, St. Louis, MO, USA).

### 4.7. Genotyping 

Chromosomal DNA for Pulsed-Field Gel Electrophoresis (PFGE) was prepared as previously described [41,42,43]. DNA was digested with *ApaI* 30 U (New England Biolabs, Beverly, MA, USA) at 25 °C for 2 h and analyzed on a Conturn-Clamped Homogenous Electric Field (CHEF DRII) [40] (Bio-Rad Laboratories, Hercules, CA, USA) with the following PFGE parameters: 20 h at 14 °C in 0.5× TBE buffer at 6 V/cm, with a forward pulse time of 5 s for 5 h, 10 s for 5 h, 22 s for 5 h, and 35 s for 5 h. A λ ladder PFGE marker was included in each run as the molecular size standard. Genetic relatedness, based on the *ApaI* PFGE profiles, was calculated by unweighted pair-group method analysis (UPGMA) [41]. *A. baumannii* strains clusters with >85% similarity were considered as belonging to the same PFGE clone [42,43].

### 4.8. Whole Genome Sequencing (WGS)

Genomic DNA was extracted using the PureLink Genomic DNA Mini Kit (Invitrogen, Waltham, MA, USA) following the manufacture’s protocol. DNA quality was evaluated using Qubit and its concentration was determined by Picogreen (Life Technologies, Carlsbad, CA, USA). Whole Genome Sequencing (WGS) was performed with the Illumina Mi-seq 300P sequencing system using paired-end (PE) read libraries prepared by Nextera XT DNA Library Preparation Kit (Illumina, San Diego, CA, USA) following the manufacture’s protocol, and the quality was evaluated as previously published [2]. The indexed libraries were quantified as previously published [2], pooled at a final concentration of 2 nM, and used for Illumina MiSeq sequencing with a PE 300 (2 × 300 bp). Raw reads were processed with QUAST (v.4.6.3) for quality evaluation, and the Cutadapter tool (v.1.16) implemented in Phyton (v.3.5.2) was employed to remove residual PCR primers, low-quality (Q_score < 30), and short reads (<150 bp). The filtered trimmed reads were used for downstream analysis. De novo genome assembly was performed with the SPAdes software (v.3.12.0) generating contig files for each sample. Post-assembly metrics were evaluated by QUAST (v.4.6.3).

### 4.9. Phylogeny and Genomic Epidemiology

The CSI Phylogeny tool was used to study the genomic relationship of strains. On the other hand, genomic epidemiology was assessed by the ResFinder (v4.1) and K-mer Resistance (v.2.2) tools to identify acquired antimicrobial resistance (AMR) genes and the known non-synonymous SNPs (nsSNPs) correlated to AMR genes using a threshold of 98% for nucleotide sequence identity and a minimum coverage of 60% [44]. Multi Locus Sequence Typing (MLST) was genomically determined using the MLST software (v1.8) [45] according to the Oxford University and Pasteur Institute databases, Mobile genetic elements (MGEs) were searched using Mobile element Finder (v1.0.3) [46] and prophages using the PHAge Search Tool (PHAST) [47] considering only the prophage regions detected with a completeness score >90. CRISPRFinder was used to identify the presence of CRISPR/Cas systems and spacers in the genomes studied. CRISPR array type was assessed using CRISPRCasdb, where CRISPR4 represents level 4 CRISPRs (the most reliable ones), while CRISPR levels 1, 2, and 3 can be considered as false CRISPRs [48]. Kaptive, a tool based on the variability of outer-core (OC) components of the lipooligosaccharide (LOS) forming a lipopolysaccharide (LPS) and K locus (KL) involved in the synthesis of the capsular polysaccharide (CPS, K, or capsule), was used to investigate the bacterial surface polysaccharide locus typing and variant evaluation [49,50].

### 4.10. Single Nucleotide Polymorphisms (SNPs)

Single Nucleotide Polymorphism (SNP) calls were carried out from the PE library raw reads as already published [2]. *A. baumannii* ACICU (CP000863.1) genome was used as the reference genome sequence for SNP mapping.

### 4.11. Genomic Single-Nucleotide Polymorphism Effect Prediction

The prediction of the whole-genome SNPs (SNPome) effects was performed by SnpEff (v.4.3T), a genomic variant annotation and functional effect prediction toolbox as previously published [51,52]. 

## Figures and Tables

**Table 1:** MICs, e-tests, COL- Population Analysis Profiles (PAP) MICs, LPS, Cell Envelope Charge, and COL-R acquisition phenotype in the COL-R *A. baumannii* strains.

	COL MIC	Most Frequent COL MIC	COL-R Induction		COL PAP VARIANT MICs		LPS Quantification	Cell Envelop Positive Charge	AA Substitution in Proteins already Associated to COL-R			
Strain	(mg/L)	(mg/L)	COL Induction	Post Induction COL MIC	Variant-1	Variant-2	(µg/mL)	COL Repulsion (%)	PmrB	LpxA	GalU	COL-R Phenotype
1R	128	-	-	-	-	-	1334.38	3.7	L208F	-	Q140L I273V	Full Resistance
2R	128	-	-	-	-	-	1698.02	8.87	R263H	-	Q140L I273V	Full Resistance
3R	128	-	-	-	-	-	2538.93	27.57	P170L A138T	N5V	I273V	Full Resistance
4R	1–64	8	NEG	-	8	64	257.11	49.5	-	-	I245T	Hetero Resistance
5R	2–16	16	NEG	-	2	128	1561.65	9.8	-	-	I245T S199A	Hetero Resistance
6R	2–128	64	POS	>256	-	-	139.93	6	A226T	-	I245T S199A	Adaptive Resistance
7R	2–128	2	NEG	-	8	64	3288.93	34.11	-	-	I245T S199A	Hetero Resistance
8R	2–32	32	POS	>256	-	-	811.66	31.7	S14P	-	I245T S199A	Adaptive Resistance
9R	1–128	4	NEG	-	64	128	2266.20	21.02	S14P	-	I245T S199A	Hetero Resistance

**Table 2:** Genomic epidemiology and characterization of COL-R *A. baumannii*.

Strain	*gPhyl* *Cluster*	*A. baumannii* MLST Pasteur Institute	*A. baumannii* MLST Oxford University	KL TYPE	OCL TYPE	MGEs	Prophage	PFGE Clone Profile			RESISTOME		
									β-Lactams	Aminoglycosides	Sulfonamides	Macrolides	Tetracycline
1R	gPhyl cluster-III	ST-187	ST-1839; ST-281	KL22	OCL3	IS26 (6 copies) IS6100 (1 copy) ISAba1 (16 copies) ISAba13 (5 copies) ISAba125 (7 copies) Tn6080 (1 copy)	vB_AbaS_TRS1	A	blaADC-25 blaOXA-23 blaOXA-82	aadA2 ant(2″)-Ia aph(3′)-VIa	sul1	-	-
2R	gPhyl cluster-III	ST-2	ST 1839	KL22	OCL3	IS26 (5 copies) IS6100 (1 copy) ISAba1 (14 copies) ISAba13 (13 copies) ISAba125 (3 copies)	vB_AbaS_TRS1 Bφ_B1251	A	blaADC-25 blaOXA-23 blaOXA-82	aac(3)-Ia aadA1 aadA2 ant(2″)-Ia aph(3′)-VIa	sul1	-	-
3R	gPhyl cluster-II	ST-2	ST-1816; ST-195	KL3	OCL1	IS26 (3 copies) ISAba1 (16 copies) ISAba24 (24 copies) IsAba26 (2 copies) IsVsa3 (1 copy) Tn2006 (1 copy)	vB_AbaS_TRS1	A	blaADC-25 blaOXA-23 blaOXA-66 blaTEM-1D	aph(3″)-Ib aph(3′)-Ia aph(6)-Id armA	Sul2	mphE msrE	tetB
4R	gPhyl cluster-II	ST-2	ST-218	KL28	OCL1	ISVsa3 (1 copy) ISAba13 (1 copy) ISAba27 (1 copy)	Bφ_B1251	A	blaADC-25 blaOXA-23 blaOXA-66	aph(3″)-Ib aph(6)-Id	-	-	tetB
5R	gPhyl cluster-I	ST-2	ST-1808; ST-348	KL9	OCL1	IS17 (1 copy) IS26 (1 copy) ISAba1 (1 copy) ISAba13 (1 copy) ISAba26 (1 copy) ISAba36 (1 copy) ISVsa3 (1 copy) ISEc29 (1 copy)		A	blaADC-25 blaOXA-66 blaOXA-72	aac(6′)-Ip aph(3″)-Ib aph(6)-Id armA	sul1 sul2	mphE msrE	tetB
6R	gPhyl cluster-I	ST-2	ST-1808; ST-348	KL9	OCL1	IS17 (1 copy) IS26 (1 copy) ISAba1 (1 copy) ISAba13 (1 copy) ISAba26 (1 copy) ISAba36 (1 copy) ISVsa3 (1 copy) ISEc29(1 copy)		A	blaADC-25 blaOXA-66 blaOXA-72	aac(6′)-Ip aph(3″)-Ib aph(6)-Id armA	sul1 sul2	mphE msrE	tetB
7R	gPhyl cluster-I	ST-2	ST-1808; ST-348	KL9	OCL1	IS17 (1 copy) IS26 (1 copy) ISAba1 (1 copy) ISAba13 (1 copy) ISAba26 (1 copy) ISAba36 (1 copy) ISVsa3 (1 copy) ISEc29(1 copy)		A	blaADC-25 blaOXA-66 blaOXA-72	aph(3″)-Ib aph(6)-Id armA	sul1 sul2	mphE msrE	tetB
8R	gPhyl cluster-I	ST-2	ST-1808; ST-348	KL9	OCL1	IS17 (1 copy) IS26 (1 copy) ISAba1 (1 copy) ISAba13 (1 copy) ISAba26 (1 copy) ISEc29 (1 copy) Tn6207 (1 copy)		A	blaADC-25 blaOXA-66 blaOXA-72	aph(3″)-Ib aph(6)-Id armA	sul1	mphE msrE	tetB
9R	gPhyl cluster-I	ST-2	ST-1808; ST-348	KL9	OCL1	IS17 (1 copy) IS26 (1 copy) ISAba1 (1 copy) ISAba13 (1 copy) ISAba26 (1 copy) ISEc29 (1 copy) Tn6207 (1 copy)		A	blaADC-25 blaOXA-66 blaOXA-72	aph(3″)-Ib aph(6)-Id armA	sul1	mphE msrE	tetB

**Table 3:** Clinical COL-R *A. baumannii*.

Strain	Hospital Wards	Source
1R	Anesthesia and Intensive care	Bronchial aspirate
2R	Anesthesia and Intensive care	Bronchial aspirate
3R	Anesthesia and Intensive care	Bronchial aspirate
4R	Anesthesia and Intensive care	Bronchial aspirate
5R	Anesthesia and Intensive care	Bronchial aspirate
6R	Anesthesia and Intensive care	Bronchial aspirate
7R	Burns Unit	Blood culture
8R	Anesthesia and Intensive care	Blood culture
9R	Orthopedics and Traumatology	Wound swab

## Data Availability

The genomic reads were deposited in the National Center for Biotechnology Information (NCBI) Genome database in the Sequence Read Archive (SRA), namely 1R and 2R under study accession no. SRP133297 (BioProject: PRJNA435581) and 3-9R under study accession no. SRP166981 (BioProject: PRJNA717714).

## References

[1] Sievert D.M., Ricks P., Edwards J.R., Schneider A., Patel J., Srinivasan A., Kallen A., Limbago B., Fridkin S., National Healthcare Safety Network (NHSN) Team and Participating NHSN Facilities (2013). Antimicrobial-resistant pathogens associated with healthcare-associated infections: Summary of data reported to the National Healthcare Safety Network at the Centers for Disease Control and Prevention, 2009–2010. Infect. Control Hosp. Epidemiol..

[2] Cafiso V., Stracquadanio S., Lo Verde F., Gabriele G., Mezzatesta M.L., Caio C., Pigola G., Ferro A., Stefani S. (2019). Colistin Resistant *A. baumannii*: Genomic and Transcriptomic Traits Acquired under Colistin Therapy. Front. Microbiol..

[3] Ilsan N.A., Lee Y.-J., Kuo S.-C., Lee I.-H., Huang T.-W. (2021). Antimicrobial Resistance Mechanisms and Virulence of Colistin- and Carbapenem-Resistant *Acinetobacter baumannii* Isolated from a Teaching Hospital in Taiwan. Microorganisms.

[4] Wand M.E., Bock L.J., Bonney L.C., Sutton J.M. (2015). Retention of virulence following adaptation to colistin in *Acinetobacter baumannii* reflects the mechanism of resistance. J. Antimicrob. Chemother..

[5] Geisinger E., Isberg R.R. (2015). Antibiotic modulation of capsular exopolysaccharide and virulence in *Acinetobacter baumannii*. PLoS Pathog..

[6] Martins-Sorenson N., Snesrud E., Xavier D.E., Cacci L.C., Iavarone A.T., McGann P., Riley L.W., Moreira B.M. (2020). A novel plasmid-encoded mcr-4.3 gene in a colistin-resistant *Acinetobacter baumannii* clinical strain. J. Antimicrob. Chemother..

[7] Sacco F., Visca P., Runci F., Antonelli G., Raponi G. (2021). Susceptibility Testing of Colistin for *Acinetobacter baumannii*: How Far Are We from the Truth?. Antibiotics.

[8] Karakonstantis S., Saridakis I. (2020). Colistin heteroresistance in *Acinetobacter* spp.: Systematic review and meta-analysis of the prevalence and discussion of the mechanisms and potential therapeutic implications. Int. J. Antimicrob. Agents.

[9] Li J., Rayner C.R., Nation R.L., Owen R.J., Spelman D., Tan K.E., Liolios L. (2006). Heteroresistance to colistin in multidrug-resistant *Acinetobacter baumannii*. Antimicrob. Agents Chemother..

[10] Rasidin R.S.M., Suhaili Z., Mohamed A.F.S., Hod R., Neela V., Amin-Nordin S. (2020). Time-kill and post-antibiotic effect of colistin at different static concentrations in in vitro *Acinetobacter baumannii*. Trop. Biomed..

[11] Andersson D.I., Nicoloff H., Hjort K. (2019). Mechanisms and clinical relevance of bacterial heteroresistance. Nat. Rev. Microbiol..

[12] Çağlan E., Nigiz Ş., Sancak B., Gür D. (2020). Resistance and heteroresistance to colistin among clinical isolates of *Acinetobacter baumannii*. Acta Microbiol. Immunol. Hung..

[13] Ezadi F., Jamali A., Heidari A., Javid N., Ardebili A. (2020). Heteroresistance to colistin in oxacillinase-producing carbapenem-resistant *Acinetobacter baumannii* clinical isolates from Gorgan, Northern Iran. J. Glob. Antimicrob. Resist..

[14] Thet K.T., Lunha K., Srisrattakarn A., Lulitanond A., Tavichakorntrakool R., Kuwatjanakul W., Charoensri N., Chanawong A. (2020). Colistin heteroresistance in carbapenem-resistant *Acinetobacter baumannii* clinical isolates from a Thai university hospital. World J. Microbiol. Biotechnol..

[15] Chen L., Lin J., Lu H., Zhang X., Wang C., Liu H., Zhang X., Li J., Cao J., Zhou T. (2020). Deciphering colistin heteroresistance in *Acinetobacter baumannii* clinical isolates from Wenzhou, China. J. Antibiot..

[16] Charretier Y., Diene S.M., Baud D., Chatellier S., Santiago-Allexant E., van Belkum A., Guigon G., Schrenzel J. (2018). Colistin Heteroresistance and Involvement of the PmrAB Regulatory System in *Acinetobacter baumannii*. Antimicrob Agents Chemother..

[17] Machado D., Antunes J., Simões A., Perdigão J., Couto I., McCusker M., Martins M., Portugal I., Pacheco T., Batista J. (2018). Contribution of efflux to colistin heteroresistance in a multidrug resistant *Acinetobacter baumannii* clinical isolate. J. Med. Microbiol..

[18] El-Sayed Ahmed M.A.E., Zhong L.L., Shen C., Yang Y., Doi Y., Tian G.B. (2020). Colistin and its role in the Era of antibiotic resistance: An extended review (2000–2019). Emerg. Microbes. Infect..

[19] Cai Y., Chai D., Wang R., Liang B., Bai N. (2012). Colistin resistance of *Acinetobacter baumannii*: Clinical reports, mechanisms and antimicrobial strategies. J. Antimicrob. Chemother..

[20] Herrera M.E., Mobilia L.N., Posse G.R. (2011). Comparative evaluation of the sensitivity of *Acinetobacter* to colistin, using the prediffusion and minimum inhibitory concentration methods: Detection of heteroresistant isolates. Rev. Argent. Microbiol..

[21] Rodriguez C.H., De Ambrosio A., Bajuk M., Spinozzi M., Nastro M., Bombicino K., Radice M., Gutkind G., Vay C., Famiglietti A. (2010). In vitro antimicrobials activity against endemic *Acinetobacter baumannii* multiresistant clones. J. Infect. Dev. Ctries..

[22] Rodriguez C.H., Bombicino K., Granados G., Nastro M., Vay C., Famiglietti A. (2009). Selection of colistin-resistant *Acinetobacter baumannii* isolates in postneurosurgical meningitis in an intensive care unit with high presence of heteroresistance to colistin. Diagn. Microbiol. Infect. Dis..

[23] Yau W., Owen R.J., Poudyal A., Bell J.M., Turnidge J.D., Yu H.H., Nation R.L., Li J. (2009). Colistin hetero-resistance in multidrug-resistant *Acinetobacter baumannii* clinical isolates from the Western Pacific region in the SENTRY antimicrobial surveillance programme. J. Infect..

[24] Barin J., Martins A.F., Heineck B.L., Barth A.L., Zavascki A.P. (2013). Hetero- and adaptive resistance to polymyxin B in OXA-23-producing carbapenem-resistant *Acinetobacter baumannii* isolates. Ann. Clin. Microbiol. Antimicrob..

[25] Cafiso V., Stracquadanio S., Lo Verde F., Dovere V., Zega A., Pigola G., Aranda J., Stefani S. (2020). COLR *Acinetobacter baumannii* sRNA Signatures: Computational Comparative Identification and Biological Targets. Front. Microbiol..

[26] Motta S.S., Cluzel P., Aldana M. (2015). Adaptive resistance in bacteria requires epigenetic inheritance, genetic noise, and cost of efflux pumps. PLoS ONE.

[27] Falagas M.E., Makris G.C., Dimopoulos G., Matthaiou D.K. (2008). Heteroresistance: A concern of increasing clinical significance?. Clin. Microbiol. Infect..

[28] Jiang S.S., Lin T.Y., Wang W.B., Liu M.C., Hsueh P.R., Liaw S.J. (2010). Characterization of UDP-glucose dehydrogenase and UDP-glucose pyrophosphorylase mutants of *Proteus mirabilis*: Defectiveness in polymyxin B resistance, swarming, and virulence. Antimicrob. Agents Chemother..

[29] Olaitan A.O., Morand S., Rolain J.M. (2014). Mechanisms of polymyxin resistance: Acquired and intrinsic resistance in bacteria. Front. Microbiol..

[30] French S., Farha M., Ellis M.J., Sameer Z., Côté J.P., Cotroneo N., Lister T., Rubio A., Brown E.D. (2020). Potentiation of Antibiotics against Gram-Negative Bacteria by Polymyxin B Analogue SPR741 from Unique Perturbation of the Outer Membrane. ACS Infect. Dis..

[31] Overview Microflex Series—Highest Performance Bench-Top MALDI-TOF, MS Bruker.com. https://www.bruker.com/products/mass-spectrometry-and-separations/maldi-toftof/microflex/overview.html.

[32] BD PhoenixTM M50 Instrument. https://www.bd.com/en-us/offerings/capabilities/microbiology-solutions/identification-and-susceptibility-testing/bd-phoenix-automated-identification-and-susceptibility-testing-system/bd-phoenix-m50-instrument.

[33] Sensititre Antimicrobial Susceptibility Testing System—IT. https://www.thermofisher.com/uk/en/home/clinical/clinical-microbiology/antimicrobial-susceptibility-testing/sensititre-antimicrobial-susceptibility-testing-system.html.

[34] Clinical and Laboratory Standards Institute (2015). Performance Standards for Antimicrobial Susceptibility Testing; 20-Third Informational Supplement.

[35] (2019). EUCASTBreakpointV_9.0. https://www.eucast.org/fileadmin/src/media/PDFs/EUCAST_files/Breakpoint_tables/v_9.0_Breakpoint_Tables.pdf.

[36] (2019). Acinetobacter baumannii: Methods and Protocols.

[37] Yang S.J., Kreiswirth B.N., Sakoulas G., Yeaman M.R., Xiong Y.Q., Sawa A., Bayer A.S. (2009). Enhanced expression of dltABCD is associated with the development of daptomycin nonsusceptibility in a clinical endocarditis isolate of *Staphylococcus aureus*. J. Infect. Dis..

[38] Yang S.J., Nast C.C., Mishra N.N., Yeaman M.R., Fey P.D., Bayer A.S. (2010). Cell wall thickening is not a universal accompaniment of the daptomycin nonsusceptibility phenotype in *Staphylococcus aureus*: Evidence for multiple resistance mechanisms. Antimicrob. Agents Chemother..

[39] Rezania S., Amirmozaffari N., Tabarraei B., Jeddi-Tehrani M., Zarei O., Alizadeh R., Masjedian F., Zarnani A.H. (2011). Extraction, Purification and Characterization of Lipopolysaccharide from *Escherichia coli* and *Salmonella typhi*. Avicenna J. Med. Biotechnol..

[40] Mezzatesta M.L., D’Andrea M.M., Migliavacca R., Giani T., Gona F., Nucleo E., Fugazza G., Pagani L., Rossolini G.M., Stefani S. (2012). Epidemiological characterization and distribution of carbapenem-resistant *Acinetobacter baumannii* clinical isolates in Italy. Clin. Microbiol. Infect..

[41] Tenover F.C., Arbeit R.D., Goering R.V., Mickelsen P.A., Murray B.E., Persing D.H., Swaminathan B. (1995). Interpreting chromosomal DNA restriction patterns produced by pulsed-field gel electrophoresis: Criteria for bacterial strain typing. J. Clin. Microbiol..

[42] Towner K.J., Levi K., Vlassiadi M., ARPAC Steering Group (2008). Genetic diversity of carbapenem-resistant isolates of *Acinetobacter baumannii* in Europe. Clin. Microbiol. Infect..

[43] McCracken M., Mataseje L.F., Loo V., Walkty A., Adam H.J., Hoban D.J., Zhanel G.G., Mulvey M.R., Canadian Antimicrobial Resistance Alliance (CARA) (2011). Characterization of *Acinetobacter baumannii* and meropenem-resistant *Pseudomonas aeruginosa* in Canada: Results of the CANWARD 2007–2009 study. Diagn Microbiol. Infect. Dis..

[44] Zankari E., Hasman H., Cosentino S., Vestergaard M., Rasmussen S., Lund O., Aarestrup F.M., Larsen M.V. (2012). Identification of acquired antimicrobial resistance genes. J. Antimicrob. Chemother..

[45] Larsen M.V., Cosentino S., Rasmussen S., Friis C., Hasman H., Marvig R.L., Jelsbak L., Sicheritz-Pontén T., Ussery D.W., Aarestrup F.M. (2012). Multilocus sequence typing of total-genome-sequenced bacteria. J. Clin. Microbiol..

[46] Johansson M.H.K., Bortolaia V., Tansirichaiya S., Aarestrup F.M., Roberts A.P., Petersen T.N. (2021). Detection of mobile genetic elements associated with antibiotic resistance in *Salmonella enterica* using a newly developed web tool: Mobile Element Finder. J. Antimicrob. Chemother..

[47] Zhou Y., Liang Y., Lynch K.H., Dennis J.J., Wishart D.S. (2011). PHAST: A Fast Phage Search Tool. Nucleic Acids Res..

[48] Pourcel C., Touchon M., Villeriot N., Vernadet J.P., Couvin D., Toffano-Nioche C., Vergnaud G. (2020). CRISPRCasdb a successor of CRISPRdb containing CRISPR arrays and cas genes from complete genome sequences, and tools to download and query lists of repeats and spacers. Nucleic Acids Res..

[49] Wick R.R., Heinz E., Holt K.E., Wyres K.L. (2018). Kaptive Web: User-friendly capsule and lipopolysaccharide serotype prediction for Klebsiella genomes. J. Clin. Microbiol..

[50] Wyres K.L., Cahill S.M., Holt K.E., Hall R.M., Kenyon J.J. (2020). Identification of *Acinetobacter baumannii* loci for capsular polysaccharide (KL) and lipooligosaccharide outer core (OCL) synthesis in genome assemblies using curated reference databases compatible with Kaptive. Microbial. Genom..

[51] Cingolani P., Platts A., Wang L.L., Coon M., Nguyen T., Wang L., Land S.J., Lu X., Ruden D.M. (2012). A program for annotating and predicting the effects of single nucleotide polymorphisms, SnpEff: SNPs in the genome of Drosophila melanogaster strain w1118; iso-2; iso-3. Fly.

[52] Cafiso V., Stracquadanio S., Lo Verde F., De Guidi I., Zega A., Pigola G., Stefani S. (2020). Genomic and Long-Term Transcriptomic Imprints Related to the Daptomycin Mechanism of Action Occurring in Daptomycin- and Methicillin-Resistant *Staphylococcus aureus* Under Daptomycin Exposure. Front. Microbiol..

